# The blocking of uPAR suppresses lipopolysaccharide‐induced inflammatory osteoclastogenesis and the resultant bone loss through attenuation of integrin β3/Akt pathway

**DOI:** 10.1002/iid3.116

**Published:** 2016-08-02

**Authors:** Yosuke Kanno, Akira Ishisaki, Mei Miyashita, Osamu Matsuo

**Affiliations:** ^1^ Faculty of Pharmaceutical ScienceDepartment of Clinical Pathological BiochemistryDoshisha Women's College of Liberal Arts97‐1 Kodo Kyo‐tanabeKyoto610‐0395Japan; ^2^ Division of Cellular Biosignal SciencesDepartment of BiochemistryIwate Medical University2‐1‐1 Nishitokuta, Yahaba‐choShiwa‐gunIwate028‐3694Japan; ^3^ Faculty of MedicineKinki University377‐2 OhnohigashiOsakasayama589‐8511Japan

**Keywords:** Inflammation, osteoclasts, uPAR

## Abstract

**Introduction:**

Chronic inflammatory diseases, such as rheumatoid arthritis and periodontitis, cause the bone destruction by promotion of the differentiation of monocyte/macrophage lineage cells into mature osteoclasts (OCs) with active bone‐resorbing character. However, the detailed mechanisms underlying this disorder remain unclear. We herein investigated the role of urokinase plasminogen activator receptor (uPAR) in the bone destruction caused by chronic inflammation.

**Methods:**

We investigated that the effect of uPAR on inflammatory OC formation induced by lipopolysaccharide (LPS) in inflammatory diseases.

**Results:**

We found that the LPS more weakly induced OC formation and the resultant bone loss in uPAR‐deficient mice than in wild‐type mice. Additionally, we demonstrated that uPAR significantly potentiated LPS‐induced OC formation of RAW264.7 mouse monocyte/macrophage linage cells in integrin β3/Akt‐dependent manner. Moreover, we showed that the blocking of uPAR function by the administration of anti‐uPAR neutralizing antibody significantly attenuated the LPS‐induced OC formation and the resultant bone loss in mice.

**Conclusions:**

These results strongly suggest that uPAR negatively regulates the LPS‐induced inflammatory OC formation and the resultant bone loss mediated through the integrin β3/Akt pathway. Our findings partly clarify the molecular mechanisms underlying bone destruction caused by chronic inflammatory diseases, and would benefit research on identifying antibody therapy for the treatment of these diseases.

## Introduction 

Chronic inflammatory diseases, such as rheumatoid arthritis (RA) and periodontitis, frequently induce the bone destruction 1. The bone destruction is caused by an increase of differentiated and activated osteoclasts (OCs), and the resultant bone resorption is physiologically regulated by the receptor activator of NF‐κB (RANK) on OCs and its ligand (RANKL) or osteoprotegerin derived from osteoblasts (OBs) in the homeostasis of bone metabolism 2. On the other hand, soluble factors including lipopolysaccharide (LPS) and pro‐inflammatory cytokines, such as tumor necrosis factor (TNF)‐α, from inflammatory tissues pathologically induce bone loss by promoting OC‐differentiation and OC‐activation in an OBs‐independent manner 3, 4, 5. Thus, inflammation perturbs bone metabolism and promotes osteoclastogenesis, and the resultant bone loss; however, the molecular mechanism underlying the inflammation‐mediated bone loss remains to be clarified. 

LPS is recognized as a pathogen of inflammatory bone destruction observed in osteomyelitis and periodontitis 6. LPS, which is the major component of the outer membrane of gram‐negative bacteria, such as *Poryphyromonas gingivalis*, induces various signals through toll‐like receptor 4 (TLR4) 7. These signals through TLR4 make macrophages, lymphocytes, and endothelial cells release proinflammatory cytokines, such as TNF‐α, IL‐1β, and IL‐6 8, 9, 10. The release of these proinflammatory cytokines promotes systemic inflammation 11 and the subsequent bone destruction 3, 4, 5. Additionally, it has been reported that LPS induces OC formation in bone marrow‐derived cells or RAW264.7 cells by itself 12, 13, 14. Clinical and epidemiological studies revealed that periodontitis is related to RA 15. Intriguingly, DNA from the periodontitis‐associated bacteria *P. gingivalis* was detected in serum and synovial fluid from patients with RA 16, suggesting that LPS from *P. gingivalis* might play important roles on initiation of chronic inflammation at joints surrounded by synovial tissues in RA patients accompanied with the articular bone destruction.

Urokinase plasminogen activator receptor (uPAR) is a glycosylphosphatydilinositol (GPI)‐anchored protein composed of three domains (D1, D2, and D3). uPAR not only functions in uPA activation and plasmin generation, but also promotes several intercellular signaling by the interaction with transmembrane proteins such as integrins, and then mediates cellular adhesion, differentiation, proliferation, migration, cell survival 17. uPAR is expressed in multiple cells, such as monocytes, macrophages, T cells 18, 19. The uPAR expression is elevated during inflammation and tissue remodeling, and is associated with the development of inflammatory diseases, such as RA, periodontitis, cancer, and fibrosis 19, 20, 21, 22, 23, 24, 25. uPAR also plays an important role in the regulation of bone homeostasis by influencing OB function and OC formation under non‐inflammatory condition 26, 27. Kalbasi Anaraki et al. 27 demonstrated that uPAR potentiates the RANK/RANKL‐induced physiological osteoclastogenesis of mouse monocyte/macrophage linage cells in a PI3K/Akt‐dependent manner. However, it remains to be clarified how uPAR affects osteoclastogenesis pathologically initiated by a chronic inflammatory agent such as LPS, and whether blocking of uPAR function attenuates the bone destruction caused by chronic inflammation.

We herein reported the roles of uPAR in the LPS‐induced inflammatory OC formation and the resultant bone loss.

## Methods

The animal experiments were approved by the Animal Research Committee of Doshisha Women's College of Liberal Arts (Approval ID: Y14‐020). All experiments were performed in accordance with relevant guidelines and regulations.

### Animals

The uPAR‐deficient (uPAR^−/−^) mice were kindly provided by Prof. D. Collen (University of Leuven, Belgium). Wild‐type mice (C57B6J) or uPAR^−/−^ mice littermates were housed in groups of two to five in filter‐top cages with a fixed 12‐h light, 12‐h dark cycle.

### Induction of bone loss by LPS in mice

LPS (25 mg/kg) was injected subcutaneously into the back of the male mice. The administration of LPS was carried out weekly for up to 4 weeks. In other studies, LPS (25 mg/kg) plus control IgG (10 μg/kg) or LPS (25 mg/kg) plus anti‐uPAR antibodies (10 μg/kg) (R&D Systems, Minneapolis, MN) was injected subcutaneously into the back of the male mice. The injection of LPS was carried out weekly for up to 4 weeks.

### Bone histology in mice

Each femur in male mice was removed, and fixed in 4% paraformaldehyde for 2 days, and then demineralized with 10% EDTA for 14 days before embedding in paraffin. Paraffin‐embedded tissue was serially sectioned at 4–7‐μm distances. Then, the expression of TRAP in each section was stained by TRAP kit (Sigma–Aldrich, St. Louis, MO).

For the quantitative evaluation of the TRAP‐positive cells number per area in the femurs of male mice, the TRAP‐stained images obtained from separate fields on the specimens were analyzed by using ImageJ 1.43u.

### Bone mineral density in mice

Bone mineral density (BMD) of femurs from mice was measured as previously described 28, 29. The BMD of femurs from mice was measured by using peripheral quantitative computed tomography with a fixed X‐ray fan beam of 50‐μm spot size, at 1 mA and 50 kVp (LaTheta LCT‐100S; Aloka, Tokyo, Japan).

### Cell culture and OC differentiation

Bone marrow‐derived cells that include a population of pre‐OCs were obtained from tibia of 5‐ to 7‐week‐old adult mice as previously described 28. Bone marrow cells and RAW264.7 mouse monocyte/macrophage lineage cells were maintained in α‐MEM supplemented with 10% fetal calf serum (FCS) and 1% penicillin‐streptomycin at 37°C in a humidified atmosphere of 5% CO_2_/95% air. Bone marrow cells or RAW 264.7 cells were cultured for 3 days with LPS (1 μg/ml) and M‐CSF (100 ng/ml) in 48‐well plates.

### Overexpression of uPAR in RAW 264.7 cells

Human uPAR plasmid (1–322) was offered from Andre Menez (CEA, Gif sur Yvette, France). RAW 264.7 cells were transfected with the uPAR expression plasmid using Lipofectamine 2000 (Invitrogen, Carlsbad, CA). From 2 day after the transfection, transfected cells were selected with 1 mg/ml G418 for 4 weeks. Then, the uPAR‐overexpressed RAW 264.7 cells were used in the following experiments.

### uPAR siRNAs study

RAW 264.7 cells were transfected with uPAR siRNA (Santa Cruz Biotechnology, Santa Cruz, CA) using Lipofectamine 2000 (Invitrogen) according to the manufacturer's instructions. A nonspecific siRNA was employed as the control.

### Western blot analysis

Western blot analysis was performed as previously described 30. We detected expressions of uPAR, TRAP, NFATc1, GAPDH, phospho‐Akt, or Akt by using anti‐uPAR antibody (Santa Cruz Biotechnology, Dallas, TX), anti‐TRAP antibody (Santa Cruz Biotechnology), anti‐NFATc1 antibody (Santa Cruz Biotechnology), anti‐GAPDH antibody (Sigma–Aldrich), anti‐phospho‐Akt antibody (Cell Signaling Technology, Danvers, MA) or anti‐Akt antibody (Cell Signaling Technology) followed incubation with horseradish peroxidase‐conjugated antibody to rabbit IgG (Amersham Pharmacia Biotech, Uppsala, Sweden).

### RNA isolation and quantitative RT‐PCR

Total RNA was extracted as previously described 28. First‐strand cDNA was synthesized from total RNA by using the High Fidelity RT‐PCR Kit (TOYOBO, Osaka, Japan). Quantitative RT‐PCR (qRT‐PCR) was performed on the IQ5 real‐time PCR detection system (Bio‐Rad, Berkeley, CA) with SYBR Green technology on cDNA generated from the reverse transcription of purified RNA. The two‐step PCR reactions were performed as 92°C for 1 sec and 60°C for 10 sec. uPAR mRNA expression was normalized against GAPDH mRNA expression using the comparative cycle threshold method. We used the following primer sequence: uPAR, 5′‐TCCACCGAATGGCTTCCAGTG‐3′ and 5′‐AGACAACACGAGGGCACACAC‐3′; GAPDH, 5′‐TGTGTCCGTCGTGGATCTGA‐3′ and 5′‐TTGCTGTTGAAGTCGCAGGAG −3′.

### Preparation of bone marrow‐derived macrophages

Bone marrow macrophages were obtained as described by Sato et al. 31. Bone marrow cells were obtained from tibia of 5‐ to 7‐week‐old adult mice, and were cultured for 16 h in α‐MEM supplemented with 10% FCS in the presence of M‐CSF (50 ng/ml). Nonadherent cells were harvested as hemopoietic cells and further cultured with M‐CSF (50 ng/ml) for 3 days. Then, the adherent cells were used as bone marrow‐derived macrophages.

### ELISA

Bone marrow‐derived macrophages or RAW 264.7 cells were cultured for 24 h with LPS (1 μg/ml). After the indicated incubation periods, the conditioned medium was collected, and the TNF‐α in the medium was then measured using a TNF‐α mouse antibody pair (Invitrogen). The absorbance of the ELISA samples was measured at 450 nm using Multiskan JX (Thermo Labsystems, Waltham, MA).

### Statistical analysis

All data are expressed as mean ± SEM. The significance of the effect of each treatment (*P* < 0.05) was determined by analysis of variance (ANOVA) followed by the least significant difference test.

## Results

### uPAR deficiency attenuates inflammatory OC formation and the resultant bone loss induced by LPS

**Figure 1:**
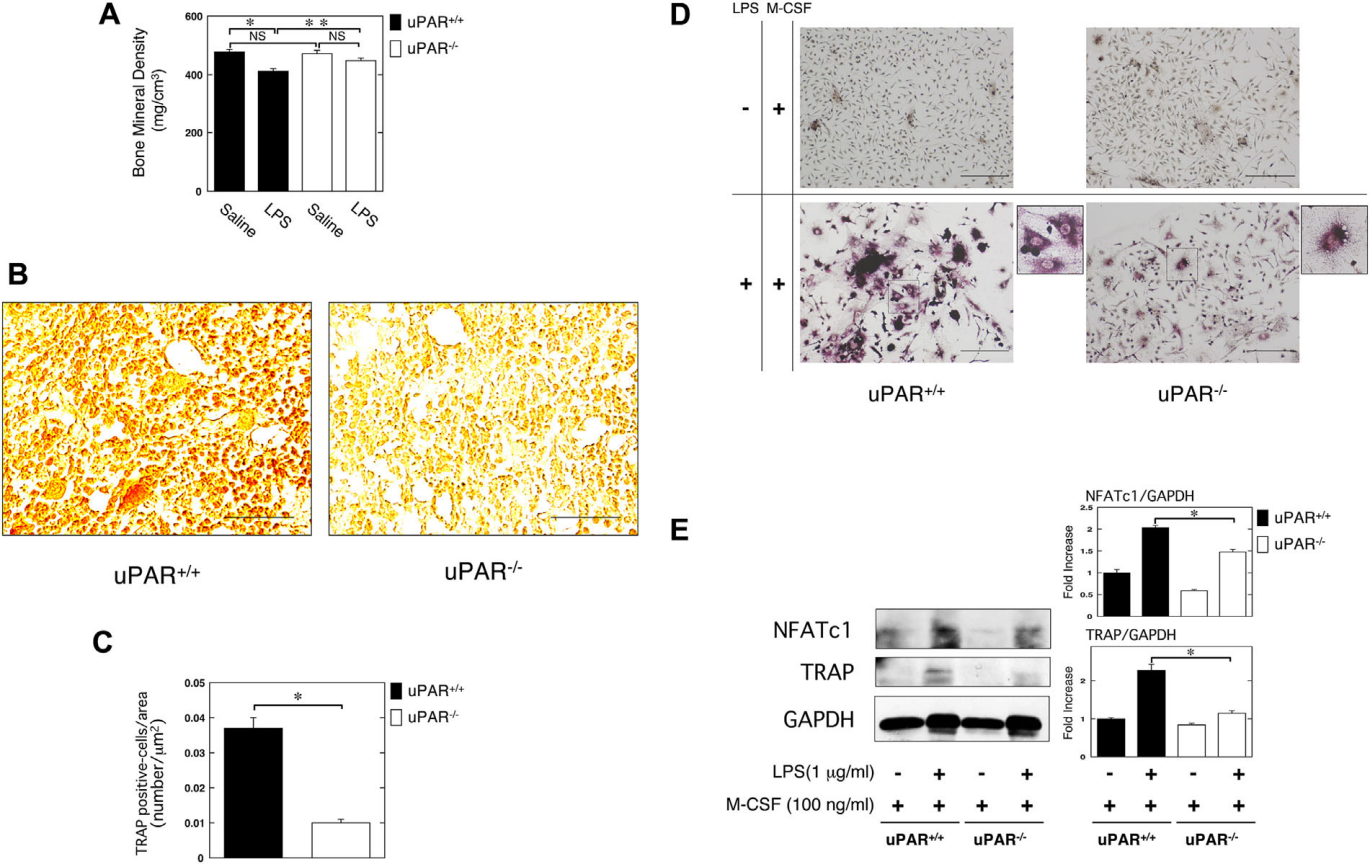
uPAR deficiency attenuates inflammatory OC formation and the resultant bone loss induced by LPS. LPS (25 mg/kg) was administered subcutaneously into the shaved back of the male mice. The administration was carried out weekly for up to 4 weeks. (A) The BMD in the femurs of the LPS‐administered male uPAR^+/+^ and uPAR^−/−^ mice was obtained from pQCT measurement (saline or LPS‐administered uPAR^+/+^ mice, *n* = 12; saline‐administered uPAR^−/−^ mice, *n* = 9; LPS‐administered uPAR^−/−^ mice, *n* = 7). (B) The TRAP‐staining of femurs in LPS‐administered male uPAR^+/+^ and uPAR^−/−^ mice. Scale bar = 50 μm. (C) The TRAP‐positive cells number per area on the decalcified sections in the LPS‐administered male uPAR^+/+^ and uPAR^−/−^ mice was quantitatively evaluated as described in the Materials and Methods section (*n* = 4). (D and E) First, bone marrow‐derived cells from the uPAR^+/+^ and uPAR^−/−^ mice were cultured for 3 days in the absence or presence of LPS (1 μg/ml) and M‐CSF (100 ng/ml). (D) TRAP‐staining was performed to detect OCs formation. Scale bar = 100 μm. The magnified image of boxed area is shown on the right of the original image. (E) The expression of TRAP and NFATc1 in bone marrow‐derived cells from the uPAR^+/+^ and uPAR^−/−^ mice was examined by a Western blot analysis. The histogram on the right panel shows quantitative representations of NFATc1 or TRAP obtained from densitometry analysis after normalization to the levels of GAPDH expression (*n* = 3). The data represent the mean ± SEM. *P < 0.01; **P < 0.05; NS, not significant.

To clarify the effects of uPAR on inflammatory OC formation and the resultant bone loss induced by LPS, we measured the BMD in the femurs of uPAR‐deficient mice following the administration of LPS. The LPS‐induced decrease of BMD in the femurs of uPAR^−/−^ mice was significantly weaker than in uPAR^+/+^ mice (Fig. 1A). We examined the status of OC formation in the femurs of mice induced by LPS administration in mice by using TRAP‐staining. The TRAP‐positive cells number per area in the femurs of uPAR^−/−^ mice was significantly less than that in the femurs of uPAR^+/+^ mice (Fig. 1B and C). It has been reported that LPS induces the differentiation to OC‐like cells form bone marrow‐derived cells or RAW264.7 cells 12, 13, 14. We evaluated OC formation in bone marrow‐derived cells from mice following the treatment of LPS and macrophage colony‐stimulating factor (M‐CSF). TRAP staining analysis revealed that uPAR deficiency attenuated LPS‐induced OC formation (Fig. 1D). Additionally, we examined the expression of OC markers NFATc1 and TRAP in the bone marrow‐derived cells from uPAR^+/+^ and uPAR^−/−^ mice at protein level. The expression of OC markers NFATc1 and TRAP in the bone marrow‐derived cells from uPAR^−/−^ mice was lower than that from uPAR^+/+^ mice (Fig. 1E).

### uPAR overexpression promotes inflammatory OC formation induced by LPS

**Figure 2:**
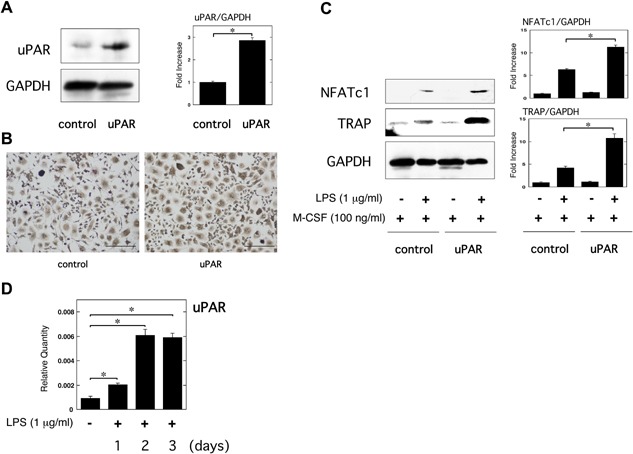
uPAR overexpression promotes inflammatory OC formation induced by LPS. (A) Overexpression of uPAR in RAW264.7 cells was confirmed by a Western blott analysis. The histogram on the right panel shows quantitative representations of uPAR obtained from densitometry analysis after normalization to the levels of GAPDH expression (*n* = 3). (B and C) First, either control or uPAR‐overexpressed RAW264.7 cells were cultured for 3 days in the absence or presence of LPS (1 μg/ml) and M‐CSF (100 ng/ml) as indicated. (B) TRAP‐staining was performed to detect OCs formation. Scale bar = 100 μm. (C) The expression of TRAP and NFATc1 in either control or uPAR‐overexpressed RAW264.7 cells was examined by a Western blot analysis. The histogram on the right panel shows quantitative representations of NFATc1 or TRAP obtained from densitometry analysis after normalization to the levels of GAPDH expression (*n* = 3). (D) RAW264.7 cells were stimulated with 1 μg/ml LPS for the indicated periods. The uPAR mRNA expression in RAW264.7 cells stimulated by LPS was evaluated by qRT‐PCR (*n* = 3). The data represent the mean ± SEM. *P < 0.01.

We first confirmed the overexpression of uPAR in RAW264.7 cells transfected with the uPAR expression plasmid (Fig. 2A). TRAP staining analysis revealed that uPAR overexpression promoted the LPS‐induced OC formation (Fig. 2B). We also found that uPAR overexpression significantly promoted the LPS‐induced NFATc1 and TRAP expression in RAW264.7 cells at protein level (Fig. 2C). Additionally, we examined the uPAR mRNA expression levels in RAW264.7 cells stimulated by LPS. qRT‐PCR analysis revealed that LPS significantly induced uPAR mRNA expression at 1–3 days after the stimulation (Fig. 2D). The maximum effect of LPS on the uPAR expression was observed at 2 days after the stimulation.

### uPAR potentiates LPS‐induced inflammatory OC formation through integrin β3/Akt pathway

**Figure 3:**
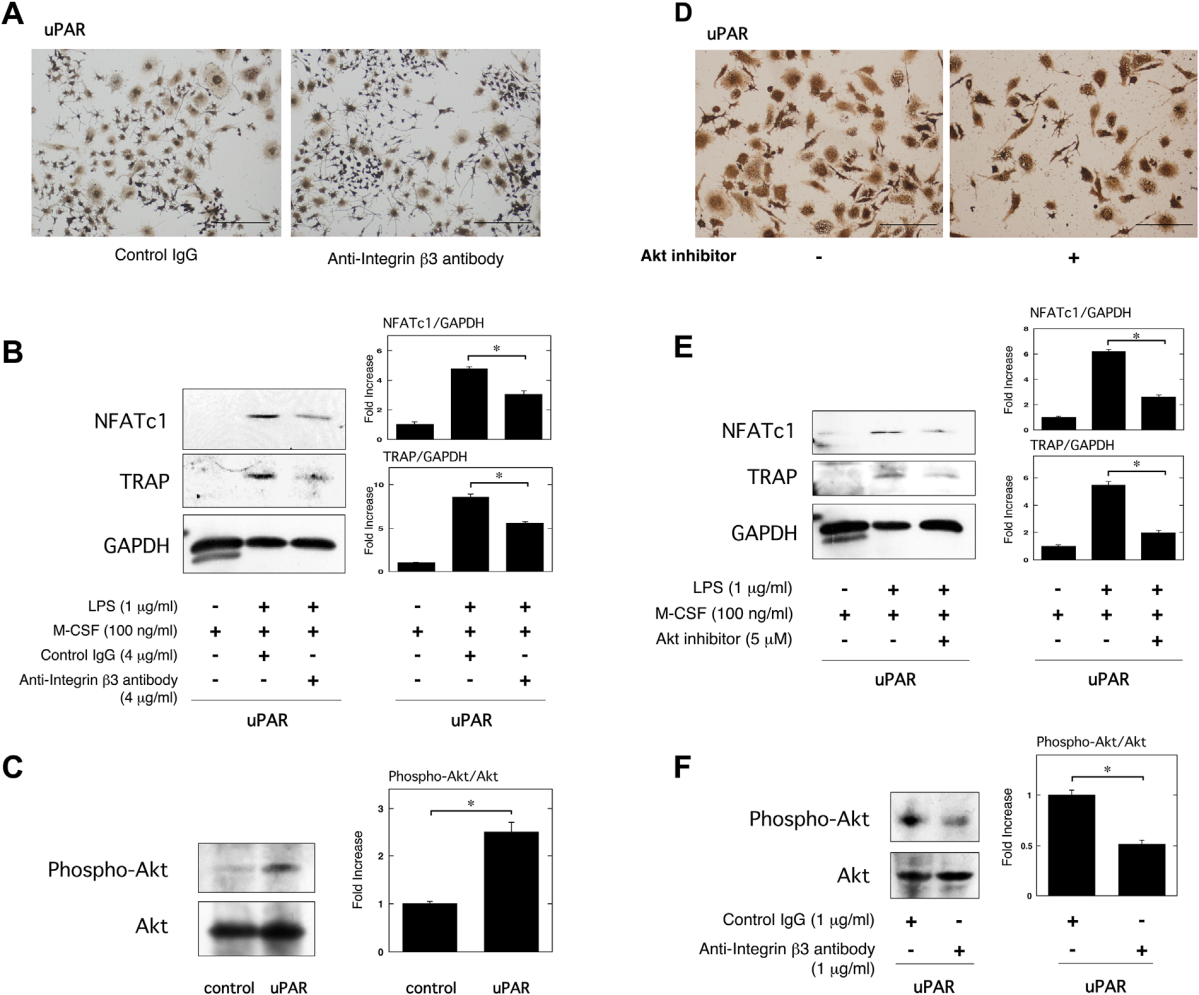
uPAR potentiates LPS‐induced inflammatory OC formation through integrin β3/Akt pathway. (A and B) First, uPAR‐overexpressed RAW264.7 cells were cultured for 3 days in the absence or presence of LPS (1 μg/ml) and M‐CSF (100 ng/ml), either control IgG (4 μg/ml) or anti‐integrin β3 neutralizing antibody (4 μg/ml) as indicated. (A) TRAP‐staining was performed to detect OCs formation. Scale bar = 100 μm. (B) The expression of TRAP and NFATc1 in uPAR‐overexpressed RAW264.7 cells was examined by a Western blot analysis. The histogram on the right panel shows quantitative representations of NFATc1 or TRAP obtained from densitometry analysis after normalization to the levels of GAPDH expression (*n* = 3). (C) Phosphorylated Akt and total Akt in either control or uPAR‐overexpressed RAW264.7 cells were examined by a Western blot analysis. The histogram on the right panel shows quantitative representations of phospho‐Akt obtained from densitometry analysis after normalization to the levels of total Akt expression (*n* = 3). (D and E) First, either control or uPAR‐overexpressed RAW264.7 cells were cultured for 3 days in the absence or presence of LPS (1 μg/ml) and M‐CSF (100 ng/ml) or Akt inhibitor (5 μM) as indicated. (D) TRAP‐staining was performed to detect OCs formation. Scale bar = 100 μm. (E) The expression of TRAP and NFATc1 in uPAR‐overexpressed RAW264.7 cells was examined by a Western blot analysis. The histogram on the right panel shows quantitative representations of NFATc1 or TRAP obtained from densitometry analysis after normalization to the levels of GAPDH expression (*n* = 3). (F) uPAR‐overexpressed RAW264.7 cells were treated with either control IgG (1 μg/ml) or anti‐integrin β3 neutralizing antibody (1 μg/ml) for 24 h. Phosphorylated Akt and total Akt were examined by a Western blot analysis. The histogram on the right panel shows quantitative representations of phospho‐Akt obtained from densitometry analysis after normalization to the levels of total Akt expression (*n* = 3). The data represent the mean ± SEM. *P < 0.01.

It has been reported that uPAR induces several intracellular signaling cascades through integrins 17, and integrin β3 regulates OC‐mediated bone resorption 32, 33. Therefore, we examined the effect of integrin β3 in the uPAR‐promoted OC formation induced by LPS. TRAP staining analysis revealed that the blocking of integrin β3 with an anti‐integrin β3 neutralizing antibody inhibited the uPAR‐promoted OC formation induced by LPS in the uPAR‐overexpressed RAW264.7 cells (Fig. 3A). We also found that the blocking of integrin β3 also significantly inhibited the uPAR‐promoted NFATc1 and TRAP expression induced by LPS in the uPAR‐overexpressed RAW264.7 cells at protein level (Fig. 3B).

In our previous studies, we have demonstrated that uPAR is associated with Akt activation 23, 34. Therefore, we examined the effect of uPAR overexpression on the status of Akt phosphorylation in RAW264.7 cells. Overexpression of uPAR clearly induced the phosphorylation of Akt in the cells (Fig. 3C). Additionally, we examined the role of Akt on the uPAR‐promoted upregulation of OC formation induced by LPS through the use of an Akt inhibitor. TRAP staining analysis revealed that Akt inhibitor abrogated the uPAR‐promoted OC formation induced by LPS in the uPAR‐overexpressed RAW264.7 cells (Fig. 3D). Akt inhibitor also significantly suppressed the uPAR‐promoted NFATc1 and TRAP expression induced by LPS in the uPAR‐overexpressed RAW264.7 cells at protein level (Fig. 3E). Moreover, we confirmed that the blocking of integrin β3 with an anti‐integrin β3 neutralizing antibody clearly suppressed the Akt phosphorylation in the uPAR‐overexpressed RAW264.7 cells (Fig. 3F).

### The knockdown of uPAR by its siRNA attenuates inflammatory OC formation induced by LPS

**Figure 4:**
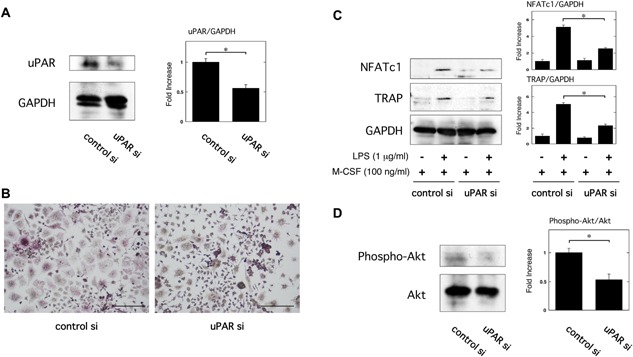
The knockdown of uPAR by its siRNA attenuates inflammatory OC formation induced by LPS. (A) Status of uPAR expression in RAW264.7 cells transfected with control or uPAR siRNA was examined by a Western blot analysis. The histogram on the right panel shows quantitative representations of uPAR obtained from densitometry analysis after normalization to the levels of GAPDH expression (*n* = 3). (B and C) First, either control or uPAR siRNA RAW264.7 cells were cultured for 3 days in the absence or presence of LPS (1 μg/ml) and M‐CSF (100 ng/ml) as indicated. (B) TRAP‐staining was performed to detect OCs formation. Scale bar = 100 μm. (C) The expression of TRAP and NFATc1 in RAW264.7 cells with control or uPAR siRNA was examined by a Western blot analysis. The histogram on the right panel shows quantitative representations of NFATc1 or TRAP obtained from densitometry analysis after normalization to the levels of GAPDH expression (*n* = 3). (D) Expression of phosphorylated Akt and total Akt in RAW264.7 cells transfected with either control or uPAR siRNA was examined by a Western blot analysis. The histogram on the right panel shows quantitative representations of phospho‐Akt obtained from densitometry analysis after normalization to the levels of total Akt expression (*n* = 3). The data represent the mean ± SEM. *P < 0.01.

We confirmed that uPAR siRNA suppressed the uPAR expression but control siRNA did not at protein level in the RAW264.7 cells (Fig. 4A). We examined the effect of downregulation of uPAR expression by siRNA on OC formation induced by LPS. TRAP staining analysis revealed that the downregulation of uPAR expression attenuated LPS‐induced OC formation in the RAW264.7 cells (Fig. 4B). The downregulation of uPAR expression also significantly suppressed LPS‐induced NFATc1 and TRAP expression in the RAW264.7 cells at protein level (Fig. 4C). Additionally, the knockdown of uPAR significantly suppressed Akt phosphorylation in the RAW264.7 cells (Fig. 4D).

### The blocking of uPAR by using anti‐uPAR neutralizing antibody suppresses inflammatory OC formation induced by LPS and the resultant bone loss in mice

**Figure 5:**
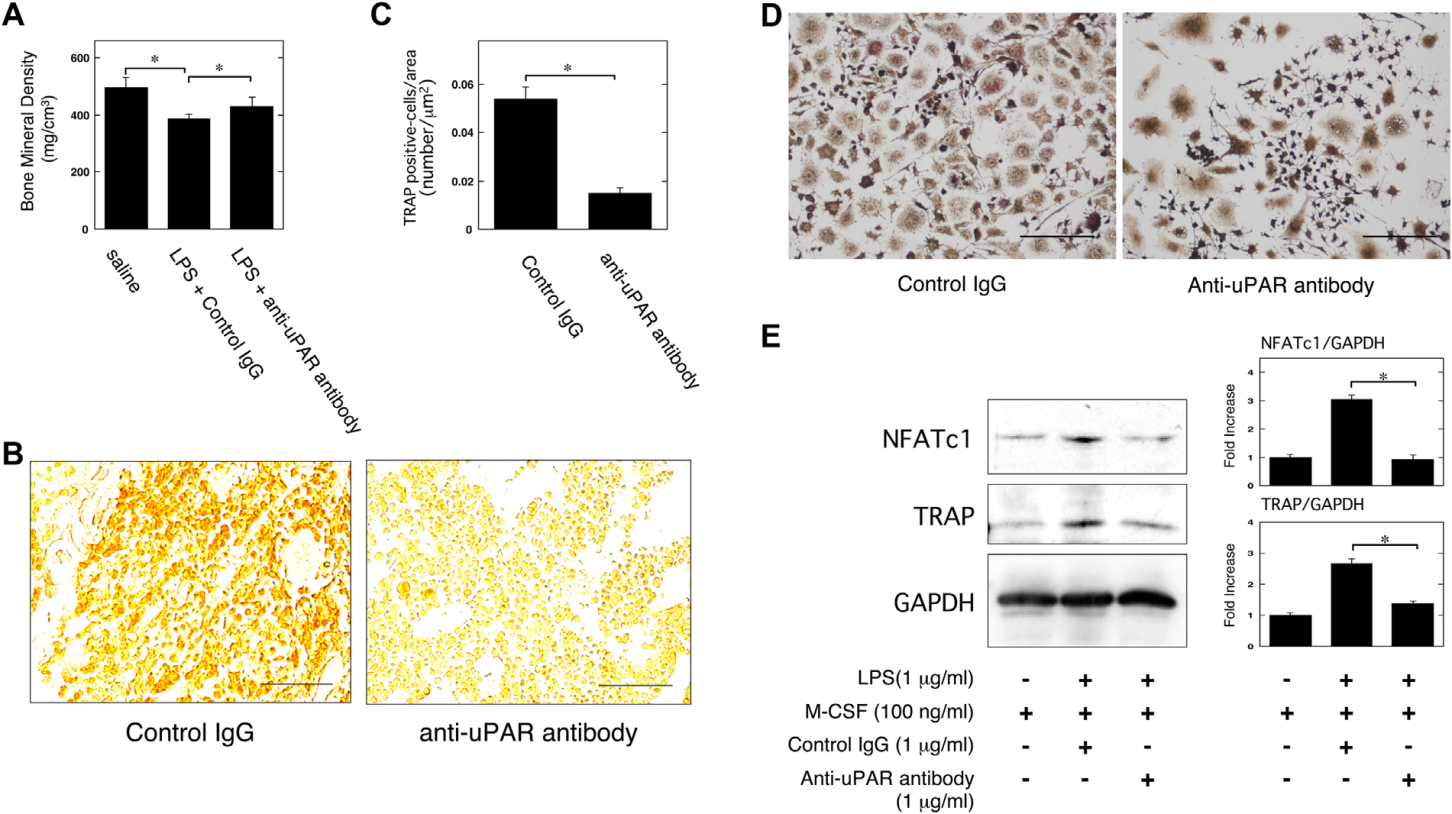
Blocking of uPAR by using anti‐uPAR neutralizing antibody suppresses inflammatory OC formation induced by LPS and the resultant bone loss in mice. (A) LPS (25 mg/kg) with either control IgG (10 μg/kg) or anti‐uPAR neutralizing antibody (10 μg/kg) was administered subcutaneously into the shaved back of the male wild‐type mice. The administration was carried out weekly for up to 4 weeks. Trabecular BMD in the femurs of the mice was obtained from pQCT measurement (saline‐administered wild‐type mice, *n* = 24; LPS plus control IgG‐administered wild‐type mice, *n* = 14; LPS plus anti‐uPAR neutralizing antibody‐administered wild‐type mice, *n* = 17). (B) The TRAP‐staining in the femurs of the male wild‐type mice treated with LPS and antibodies as described in (A). Scale bar = 50 μm. (C) The TRAP‐positive cells number per area on the decalcified sections of the male wild‐type mice treated with LPS and antibodies as described in (A) was quantitatively evaluated as described in the Materials and Methods section (*n* = 4). (D and E) First, RAW264.7 cells were cultured for 3 days in the absence or presence of LPS (1 μg/ml), M‐CSF (100 ng/ml), control IgG (1 μg/ml), or anti‐uPAR neutralizing antibody (1 μg/ml) as indicated. (D) TRAP‐staining was performed to detect OCs formation. Scale bar = 100 μm. (E) The expression of TRAP and NFATc1 in RAW264.7 cells was examined by a Western blot analysis. The histogram on the right panel shows quantitative representations of NFATc1 or TRAP obtained from densitometry analysis after normalization to the levels of GAPDH expression (*n* = 3). The data represent the mean ± SEM. *P < 0.01.

We evaluated the effect of anti‐uPAR neutralizing antibody on OC formation induced by LPS and the resultant bone loss. The subcutaneous administration of uPAR neutralizing antibody significantly attenuated the decrease of BMD (Fig. 5A) and the increase of TRAP‐positive cells induced by LPS (Fig. 5B and C). We also examined the effect of administration of uPAR neutralizing antibody on the LPS‐induced OC formation of RAW264.7 cells. TRAP staining analysis revealed that the uPAR neutralization attenuated the LPS‐induced OC formation in RAW264.7 cells (Fig. 5D). The uPAR neutralization also clearly abrogated the LPS‐induced NFATc1 and TRAP expression in RAW264.7 cells at protein level (Fig. 5E).

### The role of uPAR in the LPS‐induced TNF‐α production

**Figure 6:**
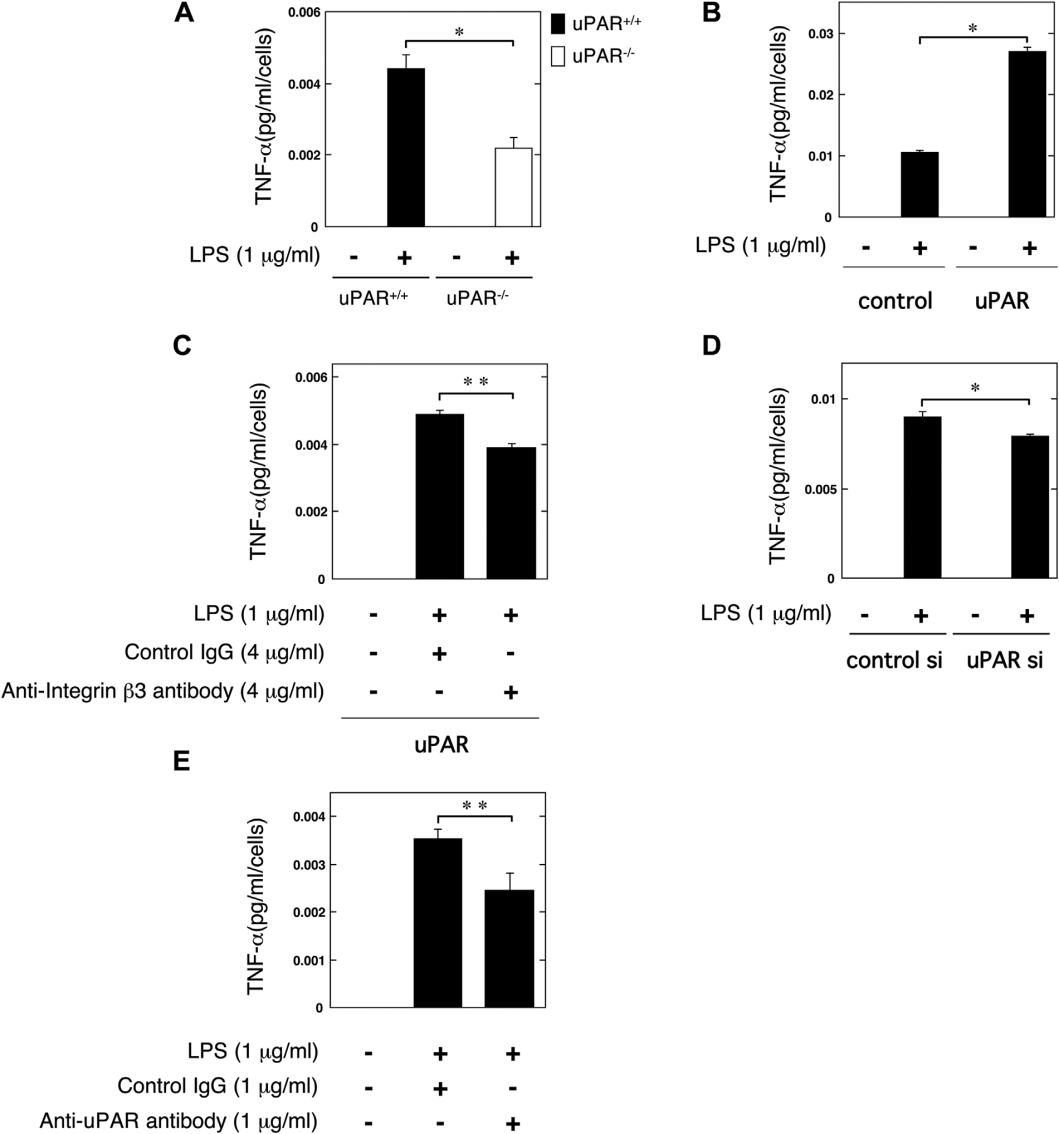
The role of uPAR on the LPS‐induced TNF‐α production during osteoclastgenesis. (A) Bone marrow‐derived macrophages from the uPAR^+/+^ and uPAR^−/−^ mice were cultured for 24 h in the absence or presence of LPS (1 μg/ml). The TNF‐α content in the conditioned media of the bone marrow‐derived macrophages from the uPAR^+/+^ and uPAR^−/−^ mice was determined by using ELISA as described in Materials and Methods section (*n* = 4). (B) Either control or uPAR‐overexpressed RAW264.7 cells were cultured for 24 h in the absence or presence of LPS (1 μg/ml). Then, the TNF‐α content in the conditioned media of the either control or uPAR‐overexpressed RAW264.7 cells was determined by using ELISA as described in Materials and Methods section (*n* = 4). (C) uPAR‐overexpressed RAW264.7 cells were cultured for 24 h in the absence or presence of LPS (1 μg/ml) with either control IgG (4 μg/ml), or anti‐integrin β3 neutralizing antibody (4 μg/ml) as indicated. The TNF‐α content in the conditioned media of the uPAR‐overexpressed RAW264.7 cells was determined by using ELISA as described in Materials and Methods section (*n* = 3). (D) RAW264.7 cells were cultured with either control or uPAR siRNA for 24 h in the absence or presence of LPS (1 μg/ml). The TNF‐α content in the conditioned media of RAW264.7 cells transfected with control or uPAR siRNA was determined by using ELISA as described in Materials and Methods section (*n* = 4). (E) RAW264.7 cells were cultured for 24 h in the presence of LPS (1 μg/ml) plus control IgG (1 μg/ml) or LPS (1 μg/ml) plus anti‐uPAR neutralizing antibody (1 μg/ml). Then, the TNF‐α content in the conditioned media of RAW264.7 cells was determined by using ELISA as described in Materials and Methods section (*n* = 3). The data represent the mean ± SEM. *P < 0.01; **P < 0.05.

LPS‐stimulated osteoclastogenesis is mediated by TNF‐α 35, 36. To clarify the role of uPAR in the LPS‐induced TNF‐α production, we examined the TNF‐α production in the bone marrow‐derived macrophages from the uPAR^+/+^ and uPAR^−/−^ mice stimulated by LPS. The TNF‐α production in the bone marrow‐derived macrophages from uPAR^−/−^ mice was significantly lower than that from uPAR^+/+^ mice (Fig. 6A). Next, we found that uPAR overexpression significantly promoted the LPS‐induced TNF‐α production in RAW264.7 cells (Fig. 6B). Additionally, the blocking of integrin β3 significantly inhibited the LPS‐induced TNF‐α production in the uPAR‐overexpressed RAW264.7 cells (Fig. 6C). Conversely, the downregulation of uPAR expression significantly attenuated the LPS‐induced TNF‐α production in RAW264.7 cells (Fig. 6D). Moreover, we showed that the uPAR neutralization significantly attenuated the LPS‐induced TNF‐α production in RAW264.7 cells (Fig. 6E).

## Discussion

Inflammation leads to OC differentiation, and then results in osteoclastogensis and bone destruction 1. LPS and proinflammatory cytokines, such as TNF‐α and IL‐1, induce OC differentiation 3, 4, 5. However, it remained to be clarified that what kinds of molecular mechanisms underlie the inflammatory OC formation in vivo. We found that uPAR‐deficient mice were protective against the inflammation‐induced osteoclastogenesis and bone loss induced by LPS (Fig. 1A–C). Additionally, the OC formation in the M‐CSF and LPS‐stimulated bone marrow‐derived cells from the uPAR‐deficient mice was significantly lower than that from wild‐type mice (Fig. 1D and E). In contrast, uPAR overexpression strengthened the LPS‐induced OC formation of RAW264.7 mouse monocyte/macrophage linage cells (Fig. 2B and C). Intriguingly, the blocking of uPAR significantly suppressed the LPS‐induced inflammatory osteoclastogenesis and bone loss in mice (Fig. 5A–C). Thus, we demonstrated, for the first time, that uPAR positively regulates OC formation induced by LPS through in vitro and in vivo studies.

RAW264.7 cells simultaneously treated with LPS and M‐CSF looked more clearly positive against TRAP staining than the cells treated with M‐CSF alone, but did not appear to be typically and predominantly enlarged multi‐nuclear cells containing more than three nuclei in each cell like as maturely differentiated OCs stimulated with RANKL and M‐CSF (Figs. 1D, 2B, 3A and D, 4B, and 5D). Additionally, simultaneous stimulation with LPS and M‐CSF more clearly induced the expression of OC markers, NFATc1 and TRAP than stimulation with M‐CSF alone (Figs. 1E, 2C, 3B and E, 4C, and 5E). These results suggest that simultaneous stimulation with LPS and M‐CSF does not induce mature OC differentiation of monocyte/macrophage lineage cells but promotes commitment of OC differentiation of the cells in vitro. It might be plausible that LPS may promote bone destruction in vivo under the influence of the other inflammatory bone reducing agents, such as TNF‐α, by inducing mature OC formation. In fact, we showed that LPS administration significantly induced osteoclastogenesis and bone loss in vivo. On the other hand, it has been reported that LPS induces OC formation in bone marrow‐derived cells or RAW264.7 cells by itself 12, 13, 14. Conversely, LPS inhibits RANKL activity by reducing the expression of RANK and M‐CSF receptor 37. These dual functions of LPS might affect the status of OC differentiation.

We demonstrated that the blocking of integrin β3 abrogated the uPAR‐caused enhancement of inflammatory OC formation induced by LPS (Fig. 3A and B). The integrin β3 is known to mediate the Akt pathway in prostate cancer cells 38, and the Akt pathway also induces OC differentiation 39, 40. We previously demonstrated that uPAR positively regulates the Akt activity in adipocytes and vascular smooth muscle cells 23, 34. Here, we showed that uPAR overexpression upregulated the Akt activity (Fig. 3C), whereas the knockdown of uPAR downregulated that (Fig. 4D). Additionally, Akt inhibitor abrogated the uPAR‐promoted enhancement of the LPS‐induced inflammatory OC formation (Fig. 3D and E). Furthermore, the blocking of integrin β3 suppressed uPAR‐caused Akt activation (Fig. 3F). Our findings demonstrated that uPAR promoted the LPS‐induced inflammatory OC formation through the integrin β3/Akt pathway.

It has been known that LPS induces the expression of uPAR in multiple cells, such as monocytes, granulocytes, lung epithelial cells, and human gingival fibroblasts 41, 42, 43, 44, 45. We herein confirmed that LPS induced the expression of uPAR in RAW264.7 cells (Fig. 2D). Additionally, the increase of uPAR expression promoted the LPS‐induced inflammatory OC formation (Fig. 2B and C), whereas the knockdown of uPAR expression inhibited that (Fig. 4B and C). These results suggest that uPAR exerts an additive effect on inflammatory OC formation induced by LPS.

It has been reported that LPS induces OC formation in bone marrow‐derived cells or RAW264.7 cells, and LPS‐induced OC formation is associated with TNF‐α production 12, 13, 14, 36. We showed that uPAR deficiency attenuated the LPS‐induced TNF‐α production in bone marrow‐derived macrophages (Fig. 6A). Additionally, uPAR knockdown by siRNA attenuated the LPS‐induced TNF‐α production in RAW264.7 cells (Fig. 6D). In contrast, uPAR overexpression strengthened the LPS‐induced TNF‐α production in RAW264.7 cells (Fig. 6B). Furthermore, the blocking of uPAR attenuated the LPS‐induced TNF‐α production (Fig. 6E). We also showed that the blocking of integrin β3 suppressed uPAR‐promoted TNF‐α production induced by LPS (Fig. 6C). Therefore, the uPAR‐regulated TNF‐α production might mediate the LPS‐induced inflammatory OC formation. However, further investigations would be required to clarify the details of relationship between the uPAR‐mediated TNF‐α production and OC formation.

Taken together, these results strongly suggest that the blocking of uPAR suppresses LPS‐induced inflammatory osteoclastogenesis and bone loss through the attenuation of integrin β3/Akt pathway. Our findings partly clarify the molecular mechanisms underlying bone destruction caused by chronic inflammatory diseases and would benefit research on identifying antibody therapy for the treatment of these diseases at the molecular level.

## Conflict of Interest

All authors state that they have no conflicts of interest.

## Author Contributions

YK conceived and designed the experiments; YK, AI, MM, OM were involved in the experiments; YK analyzed the data; YK, AI, and OM wrote the manuscript.
